# Questionable science and reproducibility in electrical brain stimulation research

**DOI:** 10.1371/journal.pone.0175635

**Published:** 2017-04-26

**Authors:** Martin E. Héroux, Colleen K. Loo, Janet L. Taylor, Simon C. Gandevia

**Affiliations:** 1 Neuroscience Research Australia, Randwick, NSW, Australia; 2 School of Medical Sciences, University of New South Wale, Sydney, NSW, Australia; 3 School of Psychiatry, University of New South Wales, Sydney, NSW, Australia; 4 Black Dog Institute, University of New South Wales Sydney, NSW, Australia; 5 Department of Psychiatry, St. George Hospital, Sydney, NSW, Australia; 6 Prince of Wales Clinical School, University of New South Wales, Sydney, NSW, Australia; Tilburg University, NETHERLANDS

## Abstract

Electrical brain stimulation (EBS) is a trendy new technique used to change brain function and treat neurological, psychiatric and psychological disorders. We were curious whether the published literature, which is dominated by positive results, reflects the experience of researchers using EBS. Specifically, we wanted to know whether researchers are able to reproduce published EBS effects and whether they engage in, but fail to report, questionable research practices. We invited 976 researchers to complete an online survey. We also audited 100 randomly-selected published EBS papers. A total of 154 researchers completed the survey. Survey respondents had a median of 3 [1 to 6, IQR] published EBS papers (1180 total) and 2 [1 to 3] unpublished ones (380 total). With anodal and cathodal EBS, the two most widely used techniques, 45–50% of researchers reported being able to routinely reproduce published results. When asked about how study sample size was determined, 69% of respondents reported using the sample size of published studies, while 61% had used power calculations, and 32% had based their decision on pilot data. In contrast, our audit found only 6 papers where power calculations were used and a single paper in which pilot data was used. When asked about questionable research practices, survey respondents were aware of other researchers who selectively reported study outcomes (41%) and experimental conditions (36%), adjusted statistical analysis to optimise results (43%), and engaged in other shady practices (20%). Fewer respondents admitted to engaging in these practices themselves, although 25% admitted to adjusting statistical analysis to optimize results. There was strong agreement that such practices should be reported in research papers; however, our audit found only two such admissions. The present survey confirms that questionable research practices and poor reproducibility are present in EBS studies. The belief that EBS is effective needs to be replaced by a more rigorous approach so that reproducible brain stimulation methods can be devised and applied.

## Introduction

Scientists agree that we are facing a crisis of confidence [1]. Research results are irreproducible, from dozens of psychology findings [2] to hundreds and even thousands of genetic [3] and fMRI [4] discoveries. Some have even argued that the majority of the published literature must be false [5]. Neuroscience, a field filled with statistically underpowered studies [6], unfortunately is at the forefront of this reproducibility crisis.

Transcranial magnetic stimulation is a popular, non-invasive and non-painful technique used by researchers and clinicians to assess and modulate brain function. Recently, we surveyed researchers on their ability to reproduce findings from studies that used transcranial magnetic stimulation to modulate non-invasively the excitability of the human motor cortex [7]. Only 40–55% of survey respondents were able to routinely reproduce previously published results. Worrisome was the finding that researchers engaged in, but failed to report, questionable research practices.

Electrical brain stimulation (EBS) is a trendy method to modify brain function that has received considerable media attention [8]. Exploding on the scene less than a decade ago, the number of EBS papers has doubled to more than 3000 in less than three years. Much cheaper to perform than magnetic stimulation, EBS is claimed to improve everything from stroke motor recovery and depression to food cravings and language acquisition. However, EBS is not without controversy. Several high-profile laboratories have been unable to reproduce previously published findings [9–12].

We were curious about whether the published literature reflects the experience of researchers using EBS. Specifically, we wanted to know whether researchers are able to reproduce published EBS effects and whether they engage in but fail to report questionable research practices.

## Materials and methods

### Online survey

To assess the use of EBS to alter human brain excitability and function, we invited corresponding authors of identified publications to complete an anonymous internet-based survey (S1 and S2 Files). The study was approved by the University of New South Wales Human Research Ethics Committee (HC13326), and was conducted in accordance with the principles expressed in the Declaration of Helsinki. As the survey was anonymous and online, written or oral consent was not obtained.

Briefly, the survey asked respondents about their area of study, the number of years they had worked with EBS, the number of published and unpublished EBS papers, and how sample sizes were determined for these studies. For unpublished papers, respondents specified the reason for the failure to publish their results. Next, we asked respondents about the types of EBS protocols they had used and, for each protocol, their ability to reproduce previously published effects. If respondents indicated they only investigated unpublished, novel effects, their responses were not considered when determining the ability of researchers to reproduce previously published results. Finally, we asked respondents how they thought other researchers performed and reported EBS studies and, using the same questions, we asked how they themselves performed and reported EBS studies. On completion of the survey, respondents were invited to provide additional comments. Then the respondents were entered into a draw, independently conducted by the local IT department, to win an iPad.

### Pubmed search and e-mail address extraction

A PubMed search was conducted on 31 December 2015 for all studies using tDCS or one of its common variants: direct current stimulation[Title/Abstract] OR tDCS[Title/Abstract] OR transcranial alternating current stimulation[Title/Abstract] OR transcranial random noise stimulation[Title/Abstract] OR HD-tDCS[Title/Abstract] OR tACS[Title/Abstract] OR transcranial electrical stimulation [Title/Abstract]. Titles and abstracts of identified references (n = 3,106) were reviewed and all human neuromodulation, brain function and clincal studies were retained. We excluded reviews, meta-analyses, errata, comments, letters, and single subject case studies as well as studies on animals, clinical trial planning, modelling electrical currents in the brain, intra-operative monitoring, and electrical stimulus perception. This resulted in a total of 1,258 references. E-mail addresses of corresponding authors and those available in the Author Information field of Pubmed references were retrieved; this resulted in 976 unique e-mail addresses and these researchers were invited to complete the survey.

### Audit of published research

A sub-sample of 100 published papers (S5 File) were selected randomly from the 1,258 identified references to determine whether the questionable research practices listed in our survey are routinely reported in publications. For each paper, we also noted: if primary study findings were positive or negative; if the Methods included a statistical analysis section; the sample size and the strategy used to determine sample size; whether error bars in figures were undefined or were standard error of the mean; whether figures included individual subject data and whether p-values of 0.1 > P > 0.05 were interpreted as statistical trends or statistically significant.

## Results

In all, 154 researchers from a variety of research disciplines completed the survey (S1 Table). Respondents had a median of 5 years [3.25 to 7.75; interquartile range] experience using EBS, and published a median of 3 [1 to 6] EBS papers (1180 total). Respondents had a median of 2 [1 to 3] unpublished EBS studies (380 total); reasons for not publishing results are presented in S2 Table.

Almost all respondents reported using anodal or cathodal transcranial direct current stimulation, whereas roughly a quarter of respondents had used transcranial alternating current stimulation, transcranial random noise stimulation or multi-channel transcranial direct current stimulation, and 5% had used pulsed transcranial direct current stimulation (Table 1). For anodal and cathodal EBS, 45–50% of respondents reported being able to routinely reproduce previously published effects (Table 1), although the size of the effect was smaller 26–27% of the time (S3 Table).

**Table 1:** Respondents’ experience with EBS protocols and ability to reproduce published findings.

	Used EBS protocol (%)	Able to reproduce published findings.		
		Yes (%)	No (%)	Sometimes (%)
AtDCS	96	50	16	35
CtDCS	81	45	26	30
tACS	27	59	20	22
tRNS	21	39	30	30
MtDCS	16	60	20	20
PtDCS	5	25	38	38

AtDCS: anodal transcranial direct current stimulation

CtDCS: cathodal transcranial direct current stimulation

tACS: transcranial alternative current stimulation

tRNS: transcranial random noise stimulation

MtDCS: multi-channel transcranial direct current stimulation

PtDCS: pulsed transcranial direct current stimulation

When asked how they determined the sample sizes of their EBS studies, 69% of respondents had used the sample size of published papers (Table 2), while 61% of respondents had previously used power calculations and 32% had based their decision on pilot data. As for the estimated number of studies for which these strategies were used, the percentages were much lower: 25% used the sample size of published papers, 26% used power calculations and only 8% used pilot data. In stark contrast to these responses, an audit of 100 randomly selected EBS papers found only 6 studies that reported power calculations and only 1 study that used pilot data to determine its sample size. All other papers failed to report how their sample size was determined.

**Table 2:** Sample size determination.

	Total studies (n [%]) *	Respondents (%) ^†^	Audit papers (%) ^‡^
Power calculation	426 [26]	61	6
Pilot data	126 [8]	32	1
Sample size from published paper	403 [25]	69	0
Personal experience	364 [22]	38	0
How data are looking	74 [5]	14	0
Stop study early—no effect	55 [3]	11	0
Stop study early—effect	21 [1]	5	0
Allow more samples to be collected	130 [8]	24	0
No strategy	41 [3]	11	93

* Respondents were asked to estimate the number of studies they conducted where they used the stated sampling strategies. Values represent total number of studies across all respondents.

^†^ Values represent percentage of respondents who reported using sampling strategy at least once; 5 respondents did not complete this question.

^‡^ Sample size across audited papers was 19 [15 to 32], median [interquartile range].

When asked about questionable research practices, survey respondents were aware of other researchers who adjusted statistical analysis to optimise results (43%) and selectively reported study outcomes (41%) and experimental conditions (36%) (Table 3). About 20% of respondents knew researchers who engaged in other shady practices (Table 3). Fewer respondents admitted to engaging in these practices themselves (Table 3), although 25% admitted to adjusting statistical analysis to optimize results.

**Table 3:** Prevalence of questionable research practices.

Questionable research practices	Others (%)	Self (%)	Audit (%)
Adjust statistical analyses in order to optimise the results	43	25	0
Not report all experimental conditions	36	13	0
Screen whether subjects are responders and not report it	21	4	0
Exclude data based on a gut feeling	21	8	0
Exclude data after looking at impact on results	20	9	0
Exclude trials or subjects without support of statistical analysis	22	8	2
Selectively report outcomes	41	14	0
Selectively report time points	18	3	0
Selectively report types of EBS used in study	12	4	0
Selectively report sub-groups of subjects	24	14	0

See S2 File for the exact wording used in the online survey.

Almost all respondents (92%) indicated that these questionable practices should be disclosed in research papers. In contrast, the audit of 100 published papers revealed only two admissions of questionable practices. Both related to the exclusion of data or subjects without the support of statistical analyses. Furthermore, 90% of audited papers reported positive primary findings, i.e. publication bias, and 30% interpreted p-values between 0.05 and 0.1 as statistical trends or statistically significant, i.e. spin [13]. In addition, few studies plotted individual subject data points in their figures so that within and between subject behaviour could be observed directly (9%) and the majority of papers (68%) erroneously used the standard error of the mean to plot data variability [14], while others failed to define the type of variability measure which was used in plots (17%).

Several researchers voiced their concerns about EBS research (S4 File):

“This field is in urgent need of both guidelines for research and clinical use, and regulations by law.”

ID217

“I think there is a huge publication bias in this field and, in my opinion, the positive results of tDCS are highly overestimated. It would have been nice to have some questions on that topic.”

ID474

“There does seem to be a suspiciously large number of positive tDCS trials published, and in almost any discipline it has been used in.”

ID31

“Although the consensus within publications in that electrical stimulation works well and is reliable, my experience of talking to other researchers at conferences and within my department suggests that there is a huge amount of unpublished, unsuccessful attempts at using the stimulation. Many of which have no clear methodological issues.”

ID583

“It would not be fair to have publication mentioning that “tDCS researchers have mentioned that are aware of other researchers that may adjust the statistics to optimize their results” or something like this. In a publish or perish academia, these practices like that are used by researchers of many fields, unfortunately. These are not specific problems for the tDCS community. I urge to be thoughtful when reporting this data.”

ID71

“I feel that a small “special group” that can publish all their research even though they have a small sample size, lack of fidelity with protocol previously registered, sub-group statistical analysis, etc. On the other hand others researchers have many difficulties to publish their works even though they followed all the requirements needed to conduct a trustful research.”

ID180

## Discussion

On the surface, EBS seems like a panacea. What other technique can claim to improve so many disparate brain functions? Warning bells have been sounded, and highlight the difficulty some research groups have reproducing published EBS effects [9–12]. Unfortunately, these concerns are largely drowned out by the never ending torrent of new papers. The present anonymous web-based survey of EBS scientists indicates that, as with transcranial magnetic stimulation, this field is not immune to issues of reproducibility, questionable research practices and publication bias.

While early EBS studies reported large, significant effects, what evidence is there that this technique is truly effective? Several meta-analyses have recently addressed this issue. For example, there is good evidence that EBS is effective in major depression [15], but not fibromyalgia pain [16], food craving and consumption [17], Parkinson’s disease [18] and stroke aphasia [19]. A common finding from these meta-analyses is that EBS studies are often of low research quality [20, 21] and that, when present, EBS effects are often small [20–24]. For example, EBS reduces chronic pain by only 12% (95% CI 8% to 15%), below the threshold for a minimal clinically important difference [22], and anodal EBS is associated with a significant reduction in reaction time, but the magnitude of this effect is small (Hedges’ g: −0.10, 95% CI −0.16 to −0.04) [24]. Importantly, these estimates exaggerate the true effect sizes because they do not take into account results from unpublished studies [25, 26].

Neuroscience research is often grossly underpowered [6], so how can so many papers report significant (i.e. p < 0.05) results when true EBS effects tend to be small? Low statistical power and publication bias may be to blame. Statistically significant effects from underpowered studies are necessarily inflated [25, 26], and often reflect false-positive results [5]. This fact explains why the first study to report an effect is often the most likely to overestimate its size (i.e., the winner’s curse) [6]. However, as more studies are published, effect sizes tend to decrease, sometimes to the point of being inconsequential. A classic example comes from transcranial magnetic stimulation research when the first paper published using a novel stimulation protocol—theta-burst stimulation—reported consistent and powerful effects in a sample of 8 subjects [27]. Years later, when the technique had been adopted by dozens if not hundreds of laboratories, the same group of researchers conducted a larger scale study involving 52 subjects; this time results were highly variable with “no overall effect” [28]. These issues are particularly troublesome because researchers continuously want to publish new discoveries. Stimulation techniques and paradigms are varied or applied to new patient groups, rendering the findings novel. Thus, many papers may suffer from the winner’s curse. Only when meta-analyses are performed and the effects of these related, but at the time novel, effects are pooled is it possible to estimate the true size of an effect. Thus, researchers using EBS must use care when designing studies. With small effects, sample sizes need to be increased to obtain adequate statistical power [6] and precise estimates of studied effects [29]. When sample size calculations are performed, they should not be based on inflated effects reported by small underpowered studies as this will result in too few subjects being tested [6].

Publication bias—where significant results are more likely to get published—was highlighted as a problem by several respondents. While our audit found 90% of papers reported significant effects for the primary research outcome, only 45–50% of respondents reported being able to routinely reproduce published effects for anodal and cathodal EBS. Even if we consider the additional 30–35% of respondents who were sometimes able to reproduce published effects, the discrepancy between the published literature and the experience of respondents likely reflects publication bias in EBS research. At the heart of publication bias is the thirst to publish novel findings and the reliance on p-values and *α* = 0.05 [30, 31]. Because statistically significant, not to be confused with scientifically or functionally significant, results are more likely to be published, practices such as p-hacking (trying several analyses and data inclusion/exclusion criteria and selectively reporting those that produce significant results) and HARKing (hypothesising after results are known) are part of the research landscape [32–34]. In our survey, for example, 25% of respondents admitted to, at one time or another, modifying their statistical analysis to obtain a favourable p-value. Other questionable practices that favour significant results in EBS research were also identified. Sadly, institutional incentives that reward the number of papers published lead to the natural selection of practices that produce significant results, and unfortunately, bad science gets results [35, 36]. In response to such issues, there have been calls to increase statistical power to 90% and decrease significance thresholds to *α* = 0.005 or 0.001 to avoid false positive results [37, 38]. With the traditional threshold of *α* = 0.05, a perfectly performed replication study has only a 50% chance of reproducing a significant effect [6, 37], a coin flip! Focus should be less on p-values and more on the scientific importance of the confidence intervals of the effects. One of the benefits of larger sample sizes is that effect size estimates are more precise [6, 29, 37], and by increasing the level of certainty surrounding the size of investigated effects, readers and editors will be interested in results regardless of their *positiveness* or *negativeness*, thus doing away with the fickle p-value [39].

Surveys can be influenced by various forms of bias. For example, those that focus on sensitive issues, questionable research practices in our case, may be biased by socially desirable responding: the tendency for respondents to give overly positive self-descriptions [40]. Unfortunately, only 0.2% of health-related surveys consider the effects of socially desirable responding on their results [41], and the present survey was not specifically designed to identify or correct for this. If present, socially desirable responding may have led us to underestimate negative practices and overestimate positive ones. However, socially desirable responding is less prevalent in anonymous self-report surveys [42], especially online ones such as ours [43]. It was recently noted that survey wording and interpretation may cause the prevalence of questionable research practices to be overestimated [44] and it is possible that this phenomenon influenced our results. Surveys are also at risk of self-selection and non-response biases [45, 46]. These biases may in part explain the glaring discrepancy between our audit and survey results. Nevertheless, the audit represents a large sample of randomly selected EBS papers and thus is a representative sample of published EBS papers. In sum, obtaining accurate estimates of questionable research practices is not simple.

The lack of transparency and scientific rigor we have uncovered likely reflects the pressure on researchers to publish significant results in high impact journals [14, 26, 35, 47–50]. This pressure drives a vicious cycle in which journals, institutions and funding agencies expect more, and, to survive and reach these expectations, scientists consciously or unconsciously adopt questionable or fraudulent research practices [7, 35, 36, 47–52]. These pressures and problems are not unique to research in EBS, nor are they new. But currently they are casting a shadow on the genuine efforts of researchers to improve brain function, a goal that is as important as ever. Fortunately, awareness of these issues is on the rise [1–7, 14, 26, 35, 36, 47–52] and recommendations and guidelines are emerging. These include justifying samples size with a priori power calculations, pre-registration of methods and analysis plans, reporting research transparently, making data and computer code openly available, and rewarding reproduction and replication studies [29, 53–59]. In EBS studies, researchers should include control brain sites in their stimulation protocols to overcome the shortcomings of sham stimulation and include control tasks to ensure the specificity of reported effects [60]. As highlighted by Poldrack et al. [55], these solutions are uncontroversial, yet their implementation is often challenging for researchers and best practices are not necessarily followed.

The clinical promise of EBS will remain illusory until the practice of neuroscience becomes more open and robust.

## Supporting information

S1 File Invitation e-mail. (PDF)

S2 File Survey. (PDF)

S3 File Survey data. (XLS)

S4 File Respondent comments. (TXT)

S5 File Audit of published papers data. (XLS)

S1 Table Respondents’ field of research. (PDF)

S2 Table Reasons why EBS study results were not published. (PDF)

S3 Table Size of effect when able to reproduce published findings and steps taken when not able to reproduce findings. (PDF)
