# Supplementary material for: Questionable science and reproducibility in electrical brain stimulation research
Source: PLoS One. 2017 Apr 26;12(4):e0175635. doi: 10.1371/journal.pone.0175635 (PMC5405934; doi:10.1371/journal.pone.0175635)
Supplement: S1 File — (PDF) [file pone.0175635.s001.pdf]

A survey on the use of electrical brain stimulation to induce cortical neuroplastic changes in humans

Dear [participant title and name],

We are writing to ask for your help in obtaining snapshot of how direct current stimulation and other forms of electrical brain stimulation (EBS) studies are being conducted and reported in the literature. We are interested in the increasing popularity of EBS as a technique to induce cortical neuroplastic changes in humans. We believe that it is timely to assess how EBS is practiced so that its use can be optimised via robust and reproducible methods. You have been identified because you were the corresponding author for a peer-reviewed publication in which EBS methods were used. We are trying to contact approximately 1000 authors.

The Survey

The survey is anonymous and consists of 8 main questions, two of which require only yes/no answers. You do not have to answer every question, and there is space in the survey for you to add any additional comments. Participation is voluntary and we will not keep track of whether or not you complete the survey. It will take approximately 5-10 minutes to complete. When submitted, your responses will be collated anonymously and stored on a secure server. **We will never be able to link your name to your responses.** We want accurate, truthful replies.

The Carrot – Win an iPad!

As a small incentive to participate, we will be raffling 1 iPads among the people that complete the survey. If you would like to participate in the raffle, you will be asked to enter an e-mail address. These e-mails will be accessible only to the head of the IT department at NeuRA, who will carry out the raffle on our behalf once the survey closes. Participating in the raffle is optional.

The Results

Once the survey closes the data will be analysed. Results from the survey will subsequently be presented at a scientific meeting and prepared for publication. We will also send a summary of our results to everyone that was invited to participate.

The Ethical Details

This project is by the Human Research Ethics Committee at the University of New South Wales (HREC HC13326). Complaints may be directed to the Ethics Secretariat, The University of New South Wales, 2052, Sydney, Australia (phone 9585 4234, fax 9385 6648, email ethics.sec@unsw.edu.au). Complaints are treated in confidence and investigated, and you will be informed of the outcome.

We welcome questions and comments about this survey, which can be done anonymously via the survey or by directly contacting us.

Yours sincerely,

Professor Simon Gandevia
MD, PhD, FAA, FRACP
s.gandevia@neura.edu.au

Associate Professor Janet Taylor
MBBS, MBiomedE, MD
j.taylor@neura.edu.au

Doctor Martin Héroux
PhD
m.heroux@neura.edu.au

Professor Colleen Loo
MBBS (Hons), FRANZCP, MD
colleen.loo@unsw.edu.au

NeuRA Discover. Conquer. Cure. BLACK DOG INSTITUTE UNSW AUSTRALIA

www.NeuRA.edu.au
Margarete Ainsworth Building
Barker Street Randwick Sydney NSW 2031 Australia
PO Box 1165 Randwick Sydney NSW 2031 Australia
T +61 2 9399 1842
