# Supplementary material for: Questionable science and reproducibility in electrical brain stimulation research
Source: PLoS One. 2017 Apr 26;12(4):e0175635. doi: 10.1371/journal.pone.0175635 (PMC5405934; doi:10.1371/journal.pone.0175635)
Supplement: S1 Table — (PDF) [file pone.0175635.s006.pdf]

S1 Table. Respondents' field of research.

	Respondents (%)*
Psychology	35
Psychiatry	18
Cognitive neuroscience	61
Motor control	38
Neurophysiology	37
Clinical neurology	22
Rehabilitation	38

* Respondents could select more than one field of research.
