# Supplementary material for: Questionable science and reproducibility in electrical brain stimulation research
Source: PLoS One. 2017 Apr 26;12(4):e0175635. doi: 10.1371/journal.pone.0175635 (PMC5405934; doi:10.1371/journal.pone.0175635)
Supplement: S2 Table — (PDF) [file pone.0175635.s007.pdf]

S2. Reasons why EBS study results were not published.

	Total studies (n [%])*	Respondents(%)†
Technical issues	48 [15]	26
Negative result – not submitted	118 [37]	53
Negative result –rejected	16 [5]	11
Positive result – not submitted	109 [34]	46
Positive result – rejected	30 [9]	18

* Respondents were asked to estimate the number of studies they had not published because of the stated reason. Values represent the total number of papers across all respondents. Although the total number of unpublished papers was 380, respondents provided reasons for 321 papers.

† Values represent percentage of respondents with unpublished results who indicated this reason **at least once.**
