# Supplementary material for: Questionable science and reproducibility in electrical brain stimulation research
Source: PLoS One. 2017 Apr 26;12(4):e0175635. doi: 10.1371/journal.pone.0175635 (PMC5405934; doi:10.1371/journal.pone.0175635)
Supplement: S3 Table — (PDF) [file pone.0175635.s008.pdf]

S3 Table. Size of effect when able to reproduce published findings and steps taken when not able to reproduce findings.

		AtDCS Respondents (%)	CtDCS Respondents (%)	tACS Respondents (%)	tRNS Respondents (%)	MtDCS Respondents (%)	PtDCS Respondents (%)
**Yes***	**Size of effect**						
	Same	67	71	70	73	58	100
	Smaller	26	27	26	27	42	0
	Larger	7	2	4	0	0	0
**No†**	**What steps were taken**						
	Publish negative results	52	26	13	11	33	50
	Stop using technique	13	35	50	44	67	25
	Contact original authors	4	6	0	0	0	0
	Modify stimulation protocol	13	6	25	22	0	25
	Test more subjects	22	13	38	11	0	25
	Select subset of responders	9	3	0	0	0	0

* Percentage relates to total number of respondents who answered 'yes' for each stimulation protocol.

†Percentage relates to total number of respondents who answered 'no' for each stimulation protocol; respondents could select more than one step.

AtDCS: anodal transcranial direct current stimulation
CtDCS: cathodal transcranial direct current stimulation
tACS: transcranial alternative current stimulation
tRNS: transcranial random noise stimulation
MtDCS: multi-channel transcranial direct current stimulation
PtDCS: pulsed transcranial direct current stimulation
